# Segmentation of phase contrast microscopy images based on multi-scale local Basic Image Features histograms

**DOI:** 10.1080/21681163.2015.1016243

**Published:** 2017-04-07

**Authors:** N. Jaccard, N. Szita, L.D. Griffin

**Affiliations:** ^a^ Department of Computer Science, University College London, London, UK; ^b^ Department of Biochemical Engineering, University College London, London, UK

**Keywords:** segmentation, phase contrast microscopy, trainable segmentation, Basic Image Features, local feature histograms, random forest

## Abstract

Phase contrast microscopy (PCM) is routinely used for the inspection of adherent cell cultures in all fields of biology and biomedicine. Key decisions for experimental protocols are often taken by an operator based on typically qualitative observations. However, automated processing and analysis of PCM images remain challenging due to the low contrast between foreground objects (cells) and background as well as various imaging artefacts. We propose a trainable pixel-wise segmentation approach whereby image structures and symmetries are encoded in the form of multi-scale Basic Image Features local histograms, and classification of them is learned by random decision trees. This approach was validated for segmentation of cell versus background, and discrimination between two different cell types. Performance close to that of state-of-the-art specialised algorithms was achieved despite the general nature of the method. The low processing time ( < 4 s per 1280 × 960 pixel images) is suitable for batch processing of experimental data as well as for interactive segmentation applications.

## 1. Introduction

Phase contrast microscopy (PCM) is widely used as the *de facto* light microscopy modality for the inspection of adherent cell cultures. PCM enables the observation of transparent cellular specimens by transforming phase shifts (induced by differences in refractive index between the sample and the surrounding medium) into changes in amplitude, which are readily detectable by the human eye or a digital camera (Zernike 1942). Automated segmentation of PCM images is made challenging by artefacts that are intrinsic to the method (Otaki 2000; ). The ‘shade-off effect’ results in low contrast between the interior of cellular objects and the image background, and bright halo artefacts around cellular objects commonly occur. Other sources of noise that can potentially interfere with PCM image segmentation include illumination patterns and non-cellular background structural noise (e.g. protein depositions and growth substrate defects).

Generic intensity thresholding approaches (e.g. Otsu's) do not usually produce satisfactory results. Specialised segmentation approaches that rely on *a priori* knowledge of the structure and properties of PCM images have been developed, including methods based on contrast filters (Bradhurst et al. 2008; Topman et al. 2011; Juneau et al. 2013; Jaccard et al. 2014), active contours (Ambühl et al. 2012; Seroussi et al. 2012), weak watershed assemblies (Debeir et al. 2008) and image formation models (Yin et al. 2012). More recently, trainable segmentation methods for microscopy images based on statistical learning of image features (e.g. intensity and texture) have been gaining traction (Kazmar et al. 2010; Yin et al. 2010; Sommer et al. 2011). Random forest classifiers (Breiman 2001) were found to be suitable to learn the patterns of features that allow correct segmentation due to their low computational complexity and their ability to accommodate large data-sets such as images (Schroff et al. 2008; Sommer et al. 2011). Typically, trainable segmentation involves using the responses to a bank of linear and nonlinear filters computed at multiple scales as feature vectors for pixel-wise classification. In Ilastik and Weka trainable segmentation (Sommer et al. 2011; Schindelin et al. 2012), two widely used software packages for trainable segmentation of biomedical images, the vector for a given pixel typically contains only a single value per scale for a given feature and thus does not fully encode potentially valuable local information and context.

In this contribution, we describe a framework for PCM image segmentation whereby local histograms encoding image features at multiple scales were used as the input to random decision trees classifiers. Unlike typical filter-based feature or patch-based representations, the use of local feature histograms leads to the discarding of local spatial structure, essentially yielding locally orderless images (Koenderink 1999). This was achieved by computing Basic Image Features (BIFs), an image representation whose pixels take one of seven values depending on local features and symmetries (Griffin et al. 2009). This small range of possible pixel values allowed efficient construction of local histograms, and classifier training was computationally tractable even in the case where multiple scales were considered. The segmentation performance is assessed using two separate PCM image data-sets which present different challenges. It is also compared with specialised PCM segmentation algorithms.

This extension of our previously published work (Jaccard et al. 2014) includes additional details on the methods used and new results including comparison with other widely used trainable segmentation software packages.

## 2. Trainable segmentation

### 2.1 General approach

**Figure 1:**
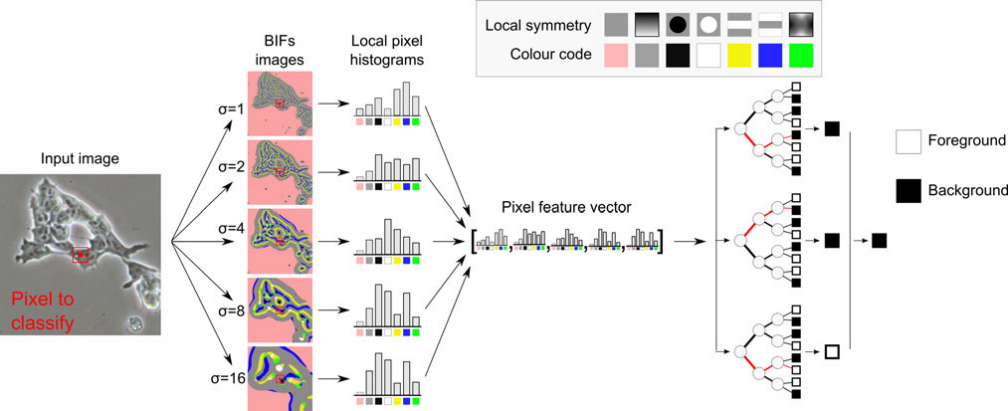
PCM pixel classification based on local histograms of BIFs. The seven BIFs and their respective colour codes are flat (i.e. no strong structure), slopes, radially symmetrical dark blobs, radially symmetrical bright blobs, dark lines, bright lines and saddle points.

PCM images were segmented based on local histograms of BIFs (Figure 1). First, BIFs of the input image were computed at various scales. Local BIFs histograms were then computed for windows centred at each pixel of the image. The feature vector for classification was constructed by concatenation of the local BIFs histograms obtained for a given pixel of the input across all scales considered (i.e. dimensions of the local structures detected). The dimensions of the pixel feature vectors were thus *M* × 7 where *M* is the number of scales considered. For comparison purposes, the situation where a single value per scale per pixel was considered, which effectively corresponded to a window diameter of 1 pixel. Pixel feature vectors for classification were then of dimensions *M* × 1.

The classifier used was a random forest with 20 trees and 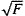 features were sampled at each split where *F* is the total number of features. The number of trees had to be chosen taking into account the balance between segmentation performance and processing time. Empirical experiments showed that increasing the number of trees above 20 only led to marginal improvements in segmentation performance while significantly increasing processing time and memory usage. The lower number of trees ensured reasonable processing times for applications where rapid feedback is required, such as interactive segmentation.

The output of the classifier was a binary label, with 1 for foreground objects (i.e. cells) and 0 for image background, which was based on the majority vote across all trees of the forest. This output was used as is for segmentation without further processing or refinement. Random forest-Matlab, an open-source implementation of Random Forest for MATLAB, was used.^1^

### 2.2 BIFs computation

**Figure 2:**
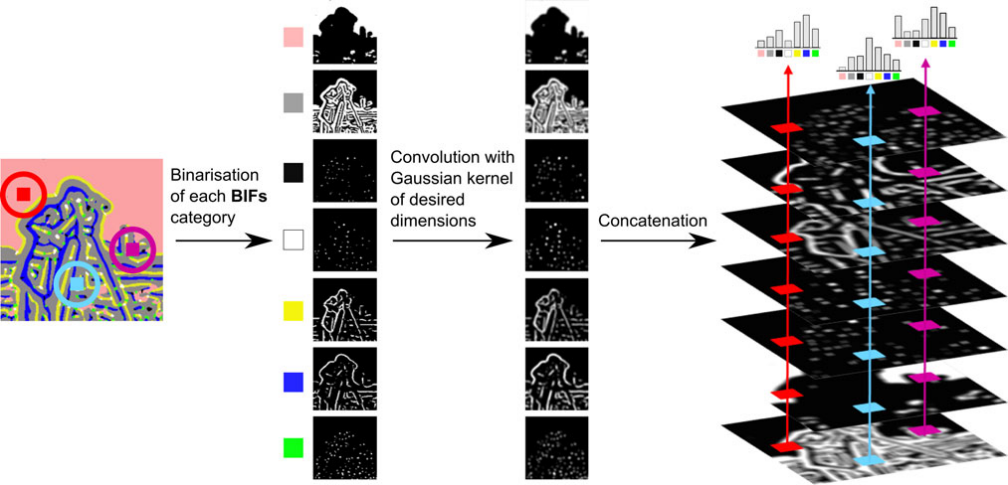
Construction of local soft-edged BIFs histograms. Cameraman image ©MIT.

The computation of BIFs consisted in classifying the output obtained from convolution of an image with a bank of derivative-of-Gaussian (DtG) filters into one of seven categories. These categories corresponded to distinct local image structures (Figure 1), as defined by local symmetries (Griffin et al. 2009): slopes, radially symmetrical dark and bright blobs, dark and bright lines, saddle points and ‘flat’ (i.e. no strong structure Figure 2).

The response of the convolution of image *I* with one of the DtG filters is denoted as 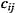 where *i* and *j* represent the order in the *x* and *y* directions, respectively. Scale-normalised response 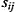 was then computed as shown in Equation (1).

(1)
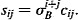

Based on the scale-normalised response, intermediate calculations are carried out as shown in Equations (2) and (3). These calculations are made for speed purposes. λ is the image Laplacian (i.e. the mean over directions of the 2nd directional derivatives) and γ is a term measuring the variance over directions of the 2nd directional derivative.

(2)
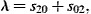

(3)
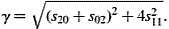

Both λ and γ were computed for each pixel of the input image *I*. Pixels were then classified in one of seven categories based on the largest of 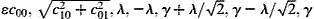, resulting in a BIFs image *I*_*B*_. BIFs computation was thus controlled by two parameters: the scale (standard deviation) σ_*B*_ of the DtG filters and a value 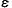 that controls when a pixel should be considered flat. For this work, 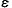 was kept at a constant value of 0.03, which was empirically found to produce good results regardless of the feature scale considered.

### 2.3 Soft-edged local BIFs histograms computation

Soft-edged local BIFs histograms were computed by convolution (Griffin et al. 2012). First, seven binary masks 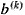 were generated as shown in Equation (4), one per BIF.

(4)
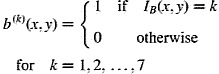

Images 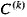 were obtained by convolution of each binary mask 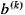 with a Gaussian kernel 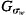 of width equal to the desired window size (*w*) and of standard deviation σ_*w*_ equal to half the window size as shown in Equation (5)

(5)
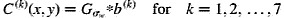

The histogram at location 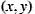 was then constructed by concatenating the values obtained across the seven 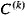 images for that location, as shown in Equation (6). The resulting histograms necessarily sum to unity.

(6)
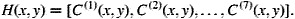

### 2.4 Intensity and contrast features

**Figure 3:**
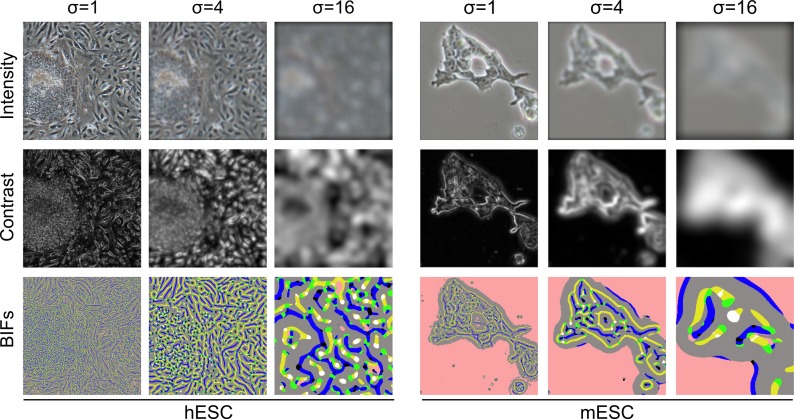
Illustration of the three types of figures considered for this work: intensity, contrast and BIFs.

In addition to BIFs, intensity and contrast features were also considered (Figure 3). For intensity features, the feature scale corresponded to the standard deviation of the Gaussian kernel used to blur the original PCM image. Contrast features were computed after application of a previously described soft-edged normalised contrast filter (Jaccard et al. 2014). The feature scale corresponded to the standard deviation of said filter. Local contrast histograms for both intensity and contrast features were constructed as described above before being downscaled to 10 bins per scale for performance reasons. Although only the best results obtained for intensity and contrast are reported in the text, the same parameter process methodology was followed for all three feature types.

### 2.5 Data-sets and segmentation performance evaluation

**Figure 4:**
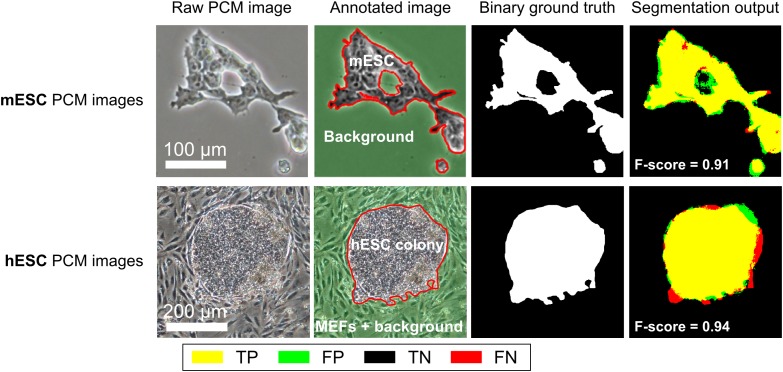
Data-sets used for segmentation performance evaluation: mESCs and hESCs PCM images. The last column shows the agreement between the segmentation output using optimal parameters and the ground truth. TP is true positives, FP is false positives, TN is true negatives and FN is false negatives.

Two data-sets were used for segmentation performance evaluation (Figure 4). The first one was a set of 50,250 × 250 pixel mouse embryonic stem cells (mESCs) PCM images (Jaccard et al. 2014). This data-set was used to evaluate the performance of the algorithm for a simple foreground versus background segmentation task. The second data-set comprised 20,500 × 500 pixel PCM images of human embryonic stem cells (hESCs) co-cultured with mouse embryonic fibroblasts (MEFs). This data-set was used to evaluate algorithm performance for the discrimination between two foreground object types with similar visual features. This second data-set was used in a previous study (Reichen et al. 2012) where a preliminary, unoptimised implementation of the approach described here-in resulted in promising results, but at the cost of long processing times (∼40 s per images). Due to the nature of the cells imaged, it was not possible to segment individual cells at a useful accuracy. Instead, the goal was the classification of pixels as either foreground or background.

Qualitative comparisons with Ilastik (version 1.1.3) and the Weka trainable segmentation plugin for FIJI (version 2.1.0) were also carried out for both data-sets. Interactive segmentation was simulated by sparse annotations of four and three full-resolution (1280 × 960) PCM images for mESCs (Figure 7) and hESCs (Figure 8) data-sets, respectively. Although all efforts were made to have equivalent annotations across all methods compared, there were slight differences at the pixel level due to differences in annotation tools. Segmentation performance for each method was evaluated on a per-pixel basis as above (using *F*-score as a metric) by comparison with fully annotated ground truth images. For Ilastik, all feature types at all scales were considered. For Weka trainable segmentation, Gaussian blur, Sobel filter, Hessian, difference of Gaussian and membrane projection features were used with sigma varying from 1.0 to 16.0. The results shown are the best obtained for each method after non-exhaustive exploration of the feature and parameter space.

The trainable segmentation scheme presented in this work was implemented in MATLAB, mainly relying on the image processing toolbox. The quoted processing times were determined using a single thread on an Intel i7-4770K CPU with 16GB of RAM and included feature computation, local histograms constructions and pixel label prediction.

## 3. Segmentation performance

**Figure 5:**
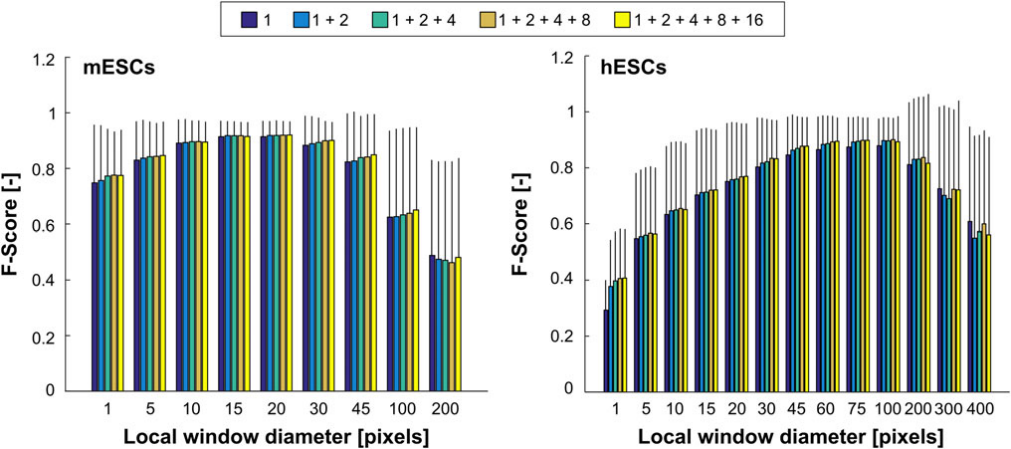
Segmentation performance in function of the local window diameter (*w*) for different combinations of BIFs scales. A window diameter of 1 pixel indicates that only a single value per scale was used. The scores shown are the mean ±  standard deviation *F*-scores obtained after LOOCV based on 50 and 20 images for mESCs and hESCs, respectively.

Segmentation performance was evaluated by comparison of the algorithm output with ground truth images annotated by human experts. The agreement between the two was calculated using the *F*-score, equivalent to Dice's coefficient (Dice 1945). A leave-one-out cross-validation (LOOCV) approach was taken whereby the classifier was trained using 50,000 pixels randomly sampled across *N* − 1 images before being used to predict the labels for each pixel of the left out image. This was repeated *N* times so that all images were left out once. The reported LOOCV *F*-score was thus the mean *F*-score across the *N* images. Segmentation performance was evaluated for both the mESC and hESC PCM images data-sets over a range of parameter values (Figure 5). The diameter of the local histogram window (*w*) was varied between 5 and 400 pixels. Up to five BIFs scales (σ_*B*_) were combined: 1, 1+2, 1+2+4, 1+2+4+8 and 1+2+4+8+16.

### 3.1 Cell versus background segmentation (mESC data-set)

The mESC data-set was employed to evaluate the performance of the proposed method for cell versus background segmentation tasks. Best performance was achieved for a local window diameter of 20 pixels and the combination of all five scales (0.92 ± 0.05). Very similar results were obtained across all combinations of scales for window diameters of 15 and 20 pixels, showing a plateau of performance around these window size values. Segmentation performance was markedly lower when using only a single value per pixel and scale, rather than local histograms. Increasing the window size beyond 20 pixels was found to be detrimental, the worst results recorded being for 200 pixel-wide windows. While single-scale schemes were usually the worst performing, increasing the number of scales only led to marginally better performance. For comparison purposes, local intensity and contrast histograms were also considered. The best performance achievable was 0.83 ± 0.12 and 0.85 ± 0.15 for intensity and contrast features, respectively.

**Figure 6:**
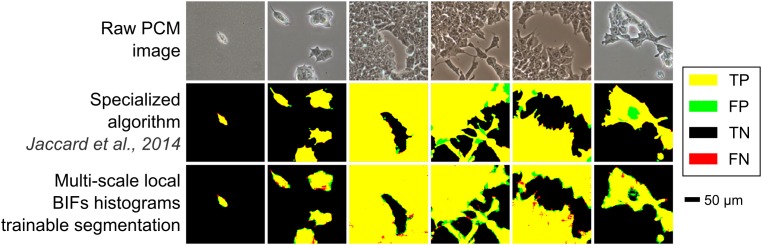
Comparison of the output from the proposed method with that of a previously described specialised PCM image segmentation algorithm. Images shown are from the mESCs data-set. Trainable segmentation based on five scales local BIFs histograms. For the specialised algorithm, images were processed using optimal segmentation parameters determined using the same mESC data-set as reported in the original paper (Jaccard et al. 2014). TP is true positives, FP is false positives, TN is true negatives and FN is false negatives.

The trainable segmentation approach (using multi-scale local BIFs histograms) was also compared with specialised PCM image segmentation algorithms based on contrast filters (Topman et al. 2011; Juneau et al. 2013; Jaccard et al. 2014). Trainable segmentation outperformed two of the three approaches and produced results approaching those obtained using the third (best performing) one (Table 1). Such results were expected as the specialised approach in question (Jaccard et al. 2014) relies on highly optimised hand-crafted algorithms taking into account known PCM image properties and structures, whereas the proposed trainable segmentation scheme relies solely on generic image features (BIFs in this case) and user-set hard constraints. Interestingly, the classifier learned how to properly label halos around foreground objects (a type of artefact intrinsic to PCM) without being explicitly designed to do so (as shown by the very few false positive pixels on the border of cellular objects in Figure 6). Holes within colonies were also accurately detected as background, which is often difficult to handle well with conventional approaches where a size threshold parameter usually dictates whether to fill the hole or not. In general, the output of the trainable segmentation algorithm was more variable than that of the specialised algorithms assessed, most likely due to using the raw output of the random forest classifier without any kind of post-processing clean-up of the segmentation mask.

**Table 1:** Comparison of the performance of the proposed trainable segmentation scheme with specialised PCM segmentation algorithms.

Method	LOOCV *F*-score
Trainable segmentation	0.92 ± 0.05
Jaccard et al. 2014	0.95 ± 0.04
Juneau et al. 2013	0.85 ± 0.10
Topman et al. 2011	0.84 ± 0.11

Notes: All results shown are mean *F*-score ±  standard deviation after LOOCV.

**Figure 7:**
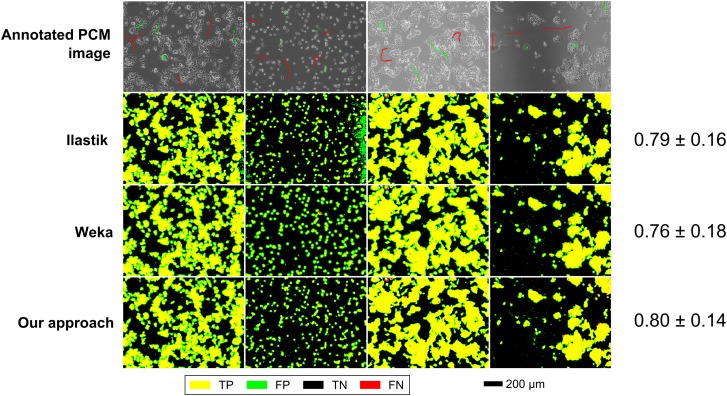
Segmentation performance of the proposed scheme (multi-scale BIFs histograms), Ilastik and Weka trainable segmentation for the mESC data-set. Numerical results are shown as mean *F*-score ± SD.

In a qualitative comparison with Ilastik and Weka segmentation in an interactive segmentation setting, the proposed scheme performed slightly better, but the difference was not significant (Figure 7). The segmentation outputs were very similar across all methods compared.

### 3.2 Discrimination between cell types in co-culture images

The ability of the proposed trainable segmentation scheme to discriminate between different cell types was assessed using PCM images of hESCs co-cultured with MEFs (Figure 4). The best segmentation performance (0.90 ± 0.08) was achieved for a local window diameter of 100 pixels and the combination of three BIFs scales (Figure 5). This optimal window diameter is significantly larger than for the mESC data-set, suggesting that pixels belonging to hESC colonies, which are mostly convex monolithic objects, were best identified over a large neighbourhood, whereas smaller window sizes were required to correctly label the comparatively smaller and more intricate features of mESCs.

Using a single feature value per pixel per scale instead of local histograms resulted in the worst performance across the conditions tested. Segmentation performance tended to increase with the local window diameter up to the aforementioned optimal 100 pixels value. A performance plateau was observed between window diameters of 60 and 100 pixels, beyond which the results rapidly deteriorated. In all cases, the combinations of at least two BIFs scales outperformed single-scale schemes. Using raw intensity and contrast features resulted in LOOCV *F*-scores of 0.71 ± 0.24 and 0.87 ± 0.10, respectively. The latter was thus close to the results obtained using BIFs.

**Figure 8:**
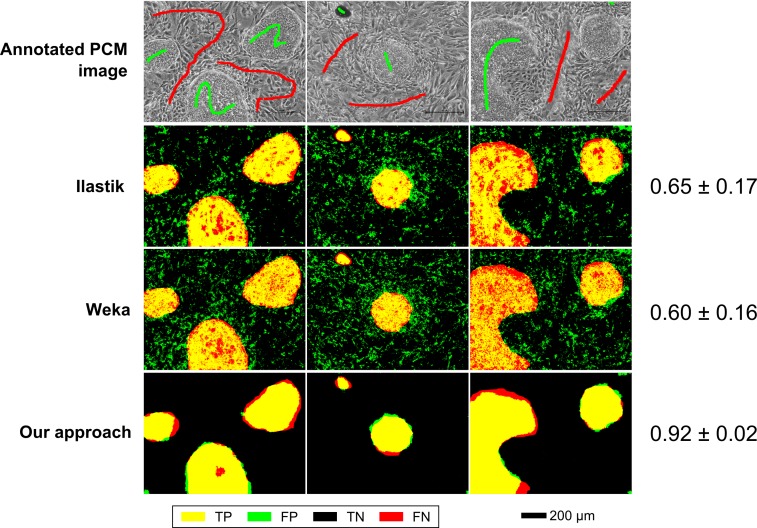
Segmentation performance of the proposed scheme (multi-scale BIFs histograms), Ilastik and Weka trainable segmentation for the hESC data-set. Numerical results are shown as mean *F*-score ± SD.

In a qualitative comparison of segmentation outputs, the proposed scheme appeared to perform significantly better than both Ilastik and Weka trainable segmentation (Figure 8). In particular, both the other software packages had a large number of false positives due to the misclassification of MEF cell pixels as hESC pixels. In contrast, the proposed scheme only had false positives at the edges of hESC colonies where the boundary between the two cell types is more ambiguous.

## 4. Summary and conclusion

In this work, we described a trainable segmentation algorithm for PCM images based on multi-scale local BIFs histograms. It performed well in foreground versus background segmentation tasks, approaching performance of state-of-the-art specialised algorithms. Indeed, the random forest classifier implicitly learned how to correct halo artefacts, which is usually done as an extra post-processing step in said algorithms (Bradhurst et al. 2008; Jaccard et al. 2014). It also produced good results for a more complex segmentation task consisting in differentiating between two types of foreground objects with similar visual attributes. The fact that two significantly different problems could be suitably addressed using the same algorithm demonstrated the versatility of trainable segmentation approaches in general, and that of the proposed method in particular. The use of local histograms was found to markedly increase segmentation performance when compared with schemes based on a single feature value per scale. In contrast, the combination of multiple BIFs scales only resulted in moderate (and in most cases not significant) improvements. In all cases, schemes using BIFs outperformed those based on intensity or contrast features. In addition, when compared with the widely used Ilastik and Weka trainable segmentation packages, the proposed multi-scale BIFs histogram scheme performed similarly for cell versus background applications and significantly better for the discrimination between two cell types.

Processing a standard microscopy image (1280 × 960 pixels) took less than 4 s using a single thread on a 3.7 GHz E5-1620 CPU, including the computations of BIFs at three scales and the construction of histograms for each pixel of the image. Using BIFs as features had the advantage of requiring only seven bin histograms per scale, which allowed their rapid computation for each pixel. It also significantly reduced the computational complexity of the offline phase (i.e. classifier training), as memory requirements and training time both increase with the number of features. In contrast, when using local 256-bin intensity feature histograms, computation time soared to more than 45 s for the same image and conditions. Specialised algorithms took on average about a second to process the same images (Jaccard et al. 2014). Interestingly, combining all feature types (intensity, contrast and BIFs) did not result in significant improvements in segmentation performance over the best results obtained with BIFs only.

These low processing times using BIFs make this method suitable for batch segmentation of large number of PCM images or that of time-lapse movie frames. To generate the results presented in this paper, the classifier was trained based on 50,000 pixels sampled across the entire data-set (minus the left out image), or less than 1.6% and 1% of the total number of pixels for the mESC and hESC data-sets, respectively. Combined with the low processing times, the ability to handle sparse annotations could enable the use of the proposed approach for interactive segmentation of PCM images.

Further improvements to the proposed scheme could include the use of multiple window sizes and BIFs scales simultaneously. This would allow the method to accommodate different applications (e.g. the cell versus background and cells versus cells scenarios shown here) without requiring additional tweaking of the window size, thus potentially increasing its robustness and generalisation. However, feature vectors of increasing size could potentially be detrimental to the processing speed without further optimisation or feature selection. Another potential venue for improvement is the use of a series architecture, whereby the first model uses features extracted from the input images only, while the subsequent models also employ the probability map obtained from the preceding model. This multi-scale context approach was previously shown to improve segmentation performance of neuron membranes in electron microscopy images (Seyedhosseini et al. 2011).

## Footnotes

^1.^ https://code.google.com/p/randomforest-matlab/. Last accessed 5 January 2015.
